# Arsenic Exposure Induces Unscheduled Mitotic S Phase Entry Coupled with Cell Death in Mouse Cortical Astrocytes

**DOI:** 10.3389/fnins.2016.00297

**Published:** 2016-06-29

**Authors:** Nang T. T. Htike, Fumihiko Maekawa, Haruka Soutome, Kazuhiro Sano, Sho Maejima, Kyaw H. Aung, Masaaki Tokuda, Shinji Tsukahara

**Affiliations:** ^1^Area of Regulatory Biology, Division of Life Science, Graduate School of Science and Engineering, Saitama UniversitySaitama, Japan; ^2^Center for Health and Environmental Risk Research, National Institute for Environmental StudiesTsukuba, Japan; ^3^Area of Life-NanoBio, Division of Strategy Research, Graduate School of Science and Engineering, Saitama UniversitySaitama, Japan; ^4^Department of Cell Physiology, Faculty of Medicine/Graduate School of Medicine, Kagawa UniversityKagawa, Japan

**Keywords:** astrocytes, sodium arsenite, cell cycle, cell death, live imaging

## Abstract

There is serious concern about arsenic in the natural environment, which exhibits neurotoxicity and increases the risk of neurodevelopmental disorders. Adverse effects of arsenic have been demonstrated in neurons, but it is not fully understood how arsenic affects other cell types in the brain. In the current study, we examined whether sodium arsenite (NaAsO_2_) affects the cell cycle, viability, and apoptosis of *in vitro*-cultured astrocytes isolated from the cerebral cortex of mice. Cultured astrocytes from transgenic mice expressing fluorescent ubiquitination-based cell cycle indicator (Fucci) were subjected to live imaging analysis to assess the effects of NaAsO_2_ (0, 1, 2, and 4 μM) on the cell cycle and number of cells. Fucci was designed to express monomeric Kusabira Orange2 (mKO2) fused with the ubiquitylation domain of hCdt1, a marker of G1 phase, and monomeric Azami Green (mAG) fused with the ubiquitylation domain of hGem, a marker of S, G2, and M phases. NaAsO_2_ concentration-dependently decreased the peak levels of the mAG/mKO2 emission ratio when the ratio had reached a peak in astrocytes without NaAsO_2_ exposure, which was due to attenuating the increase in the mAG-expressing cell number. In contrast, the mAG/mKO2 emission ratio and number of mAG-expressing cells were concentration-dependently increased by NaAsO_2_ before their peak levels, indicating unscheduled S phase entry. We further examined the fate of cells forced to enter S phase by NaAsO_2_. We found that most of these cells died up to the end of live imaging. In addition, quantification of the copy number of the glial fibrillary acidic protein gene expressed specifically in astrocytes revealed a concentration-dependent decrease caused by NaAsO_2_. However, NaAsO_2_ did not increase the amount of nucleosomes generated from DNA fragmentation and failed to alter the gene expression of molecules relevant to unscheduled S phase entry-coupled apoptosis (p21, p53, E2F1, E2F4, and Gm36566). These findings suggest that NaAsO_2_ adversely affects the cell cycle and viability of astrocytes by inducing unscheduled S phase entry coupled with cell death that may be caused by mechanisms other than apoptosis.

## Introduction

Several environmental chemicals are suspected to exert deleterious effects on development of the brain, which may result in an increased risk of neurodevelopmental disorders such as autism, attention-deficit hyperactivity disorder, and cerebral palsy (Grandjean and Landrigan, 2006, 2014). Arsenic is one of these environmental toxicants that disrupt brain development. According to a long-term prospective study of survivors of arsenic poisoning from the Morinaga milk incident in Japan, arsenic poisoning during infancy leads to a risk of mortality from neurological diseases in adulthood (Tanaka et al., 2010). Epidemiological studies suggest that chronic consumption of arsenic-contaminated water causes a reduction in the cognitive performance of school-age children (Calderon et al., 2001; Tsai et al., 2003; Wasserman et al., 2007). Thus, arsenic exposure via drinking water may be a risk factor for neurodevelopmental disorders.

Animal studies support the notion that developmental exposure to arsenic increases the risk of neurodevelopmental disorders. Exposure to sodium arsenite (NaAsO_2_), an inorganic arsenical compound, via drinking water during gestational and/or postnatal periods causes impairment of spatial learning and memory, neuromotor reflex alteration, and spontaneous locomotor deficits in adult rats (Rodriguez et al., 2002; Xi et al., 2009). Offspring of female mice, which had been chronically exposed to NaAsO_2_ via drinking water, display depression-like behavior (Martinez et al., 2008), and exhibit deficits in a hippocampus-dependent learning tasks (Martinez-Finley et al., 2009) during adulthood. Thus, NaAsO_2_ exposure during developmental periods and the associated adverse effects on brain development induce behavioral abnormalities, although the toxic mechanisms of arsenic remain to be elucidated.

*In vitro* studies of neurons have revealed that NaAsO_2_ induces apoptotic cell death in primary cultured neurons (Namgung and Xia, 2001; Wong et al., 2005) and neuronal cell lines (Koike-Kuroda et al., 2010; Keim et al., 2012). In addition, NaAsO_2_ disrupts neuritogenesis in primary cultured neurons (Maekawa et al., 2013) and neuronal cell lines (Frankel et al., 2009; Aung et al., 2013). We previously reported that NaAsO_2_-induced apoptosis is mediated by activation of caspase-3 (Koike-Kuroda et al., 2010), and that inhibition of neuritogenesis by NaAsO_2_ is caused by alterations in the expression of cytoskeletal genes tau, tubulin, and neurofilament (Aung et al., 2013), and suppression of glutamate AMPA receptor expression (Maekawa et al., 2013). The toxic mechanisms by which developmental exposure to NaAsO_2_ impairs the aforementioned brain functions and behaviors remain to be uncovered. However, based on *in vitro* studies of neurons, inorganic arsenic adversely affects the fate and maturation processes of young neurons, which may lead to abnormal formation of the neural circuits responsible for the brain functions and behaviors.

In addition to neurons, there may be other target cells of arsenic in the developing brain. Astrocytes are the largest population of glial cells, which are more abundant in the brain compared with neurons, and contribute to the formation and maintenance of the blood–brain barrier (BBB). The BBB is composed of endothelial cells, which line capillary blood vessels and connect to each other via tight junctions, and astrocytes surrounding blood capillaries via their end feet (Abbott, 2002). The BBB is not considered as a perfect barrier, although it contributes to protection of the brain against circulating xenobiotics that disrupt brain functions. The developing brain is considered to be vulnerable to toxic chemicals compared with the adult brain. One of the reasons is that the immature BBB during early development provides only partial protection against entry of chemicals into the brain (Zheng et al., 2003). Arsenite and arsenate are transferred to offspring through the placenta of pregnant mice that are exposed via drinking water, and arsenic species easily crossing the immature BBB accumulate in the brains of newborn offspring (Jin et al., 2006). Astrocytes are therefore the first brain cells that appear to be targeted by inorganic arsenic when it is transferred from the blood to the brain. Arsenite inhibits glutamate metabolism in astrocytes by reducing the activity and expression of glutamine synthase and glutamate transporters (Zhao et al., 2012). Synapse formation of primary cultured neurons is impaired by culture in conditioned medium from arsenite-exposed astrocytes (Wang et al., 2013). Taken together, the neurotoxicity of inorganic arsenic may be, at least in part, caused by its effects on astrocytes.

During brain development, neuron generation occurs first, followed by the generation of glial cells. In the cerebral cortex of rodents, astrocyte generation begins on embryonic day 18 following neurogenesis during embryonic days 12–18, and the number of astrocytes peaks in the neonatal period (Miller and Gauthier, 2007). It is assumed that neurotoxicant exposure during the developmental period affects not only neurogenesis but also the generation and proliferation of astrocytes, followed by altering the cell numbers. A reduced number of cortical glial cells is related to the pathological changes of schizophrenia and depression, indicating a causal link between glial cell abnormalities and psychiatric disorders (Cotter et al., 2001). In primary cultured rat astrocytes, inorganic arsenic decreases cell viability and increases DNA damage (Catanzaro et al., 2010). Such toxic effects of arsenite are stronger than those of arsenate (Jin et al., 2004). However, the mechanisms by which inorganic arsenic reduces the viability of astrocytes are largely unknown. Fluorescent ubiquitination-based cell cycle indicator (Fucci), which consists of monomeric Kusabira Orange2 (mKO2) fused with the ubiquitylation domain of human Cdt1 to monitor G1 phase and monomeric Azami Green (mAG) fused with the ubiquitylation domain of human Geminin to monitor S/G2/M phases, is useful to visualize the dynamics of cell cycle progression (Niwa et al., 1991; Sakaue-Sawano et al., 2008). In this study, we carried out live imaging analysis of primary cultured astrocytes originating from the cerebral cortex of Fucci transgenic (tg) mice to determine whether NaAsO_2_ exposure decreases cell viability by affecting the cell cycle. Additionally, we examined the effects of NaAsO_2_ exposure on the viability, apoptotic cell death, and expression of genes related to the cell cycle and apoptosis in cultured cortical astrocytes.

## Materials and methods

### Animals

Fucci tg mice were bred and maintained at the National Institute for Environmental Studies (Tsukuba, Japan). Wild-type C57BL/6J mice (Sankyo Labo Service, Tokyo, Japan) were bred at Saitama University (Saitama, Japan). They were housed under a controlled temperature (23 ± 2°C) and photoperiod (12:12, light:dark) with free access of tap water and standard chow. Animal procedures were conducted according to the approval and guidelines of Animal Care and Use Committee at the National Institute of Environmental Studies and Saitama University.

### *In vitro* culture of astrocytes

Fucci tg and C57BL/6J mice were sacrificed on postnatal days 1 or 2 (postnatal day 1 = day of birth). After isolation of the cerebral cortex from the brain, the meninges were removed and the cortex tissue were placed in ice-cold Dulbecco's modified Eagle's medium (Sigma-Aldrich, St. Louis, MO, USA) supplemented with 3.7 mg/L sodium bicarbonate (Sigma-Aldrich), 10 mL/L antibiotic antifungal solution (Sigma-Aldrich), which contained 10,000 units penicillin, 10 mg streptomycin, and 25 μg amphotericin B per mL, and 10% fetal bovine serum (FBS; Gibco-Invitrogen, Carlsbad, CA, USA), which is hereafter referred to as culture medium (pH 7.2). Cells were isolated from the cerebral cortex in fresh culture medium by gentle mechanical trituration, seeded on poly-L-ornithine (15 μg/L)-precoated culture dishes or plates, and maintained in culture medium at 37°C in a humidified atmosphere with 5% CO_2_. The culture medium was changed at 3 days after seeding, followed by medium changes at intervals of 3–4 days.

Cells obtained from Fucci tg mice were seeded on a four-compartment glass bottom dish (Greiner Bio-One, Kremsmünster, Austria) at 1 × 10^5^ cells per compartment (1.9 cm^2^), cultured for more than 1 week, and then applied to fluorescence microscopy (see Section Fluorescence Microscopy of Fucci-Expressing Astrocytes) and live imaging analyses (see Section Time-Lapse Analysis of Fucci-Expressing Astrocytes). Cells obtained from C57BL/6J mice were seeded on a 6-well culture plate (Asahi Glass, Tokyo, Japan) at 3 × 10^5^ cells per well (9.4 cm^2^) and cultured until 90–100% confluence. The cells were re-seeded onto new culture plates at 1 × 10^5^ cells per well after trypsinization and then used to analyze the protein expression of glial fibrillary acidic protein (GFAP), a marker of astrocytes (see Section Analysis of GFAP Immunoreactivity), and the GFAP gene copy number (see Section Analysis of GFAP Gene Copy Numbers). For gene expression analysis (see Section Analysis of mRNA Levels), the cells were re-seeded at 4 × 10^5^ cells per well after trypsinization. For apoptosis analysis, the cells were re-seeded on a 96-well culture plate (Asahi glass) at 5 × 10^3^ cells per well (see Section Apoptosis Assay). All culture dishes and plates were precoated with poly-L-ornithine (15 μg/L) before use.

### Analysis of GFAP immunoreactivity

To check the purity of cells in primary culture, we performed immunocytochemistry of GFAP. Cultured cells fixed with 4% paraformaldehyde were reacted with a polyclonal rabbit anti-GFAP antibody (1:500; Dako, Glostrup, Denmark) at 4°C overnight and then Alexa Fluor 647 goat anti-rabbit IgG (1:400; Life Technology, Carlsbad, CA, USA) for 30 min at room temperature. 4,6-Diamidino-2-phenylindole (DAPI) staining was performed to count the total cell number.

### Fluorescence microscopy of fucci-expressing astrocytes

To determine the cell cycle duration of astrocytes, we observed astrocytes from Fucci tg mice under a fluorescence microscope (BioZero 8100; Keyence, Osaka, Japan) equipped with an mKO2 filter (excitation filter: 542AF15; emission filter: 585QM30; dichroic mirror: 560 DRLP; Opto science, Tokyo, Japan) and a mAG filter (excitation filter: 475QM20; emission filter: 518QM32; dichroic mirror: 500DRLP; Opto science). First, the cell cycle of each astrocyte was synchronized by serum starvation. Culturing in medium supplemented with a low concentration of FBS (0.5%) for 72 h is effective to increase the population of cells at G1 phase (Khammanit et al., 2008). Therefore, 30–40% confluent astrocytes were incubated in culture medium supplemented with 0.1% FBS for 3 days. The astrocytes were again incubated in culture medium containing 10% FBS, and fluorescence images of mKO2 and mAG were captured every 6 h for 150 h using an objective lens (Plan Fluor ELWD DM 20 × C, NA 0.45; Nikon, Tokyo, Japan) and a CCD camera in the BioZero 8100 fluorescence microscope. In each culture, digital images were obtained in three regions (0.58 mm^2^/region, 1.74 mm^2^ in total) that were randomly selected in the culture dish. Fluorescence microscopy was performed in four primary cultures derived from different animals.

Image analysis of mKO2 and mAG expression was performed with BZ-II analyzer software (Keyence). The digital images were modified to remove the background signal, and the red-green-blue (RGB) digital images of mKO2 and mAG were converted to monochromatic color images (red, mKO2; green, mAG). The monochromatic color images of mKO2 and mAG were merged at each time point to determine the intensities of mKO2 and mAG expression in the same region, which were obtained by measuring the brightness of the red and green signals, respectively. After measuring the brightness values of red and green signals in the same region of each merged image, the mAG/mKO2 emission ratio was calculated by dividing the brightness value of the green signal by that of the red signal. The mAG/mKO2 emission ratio at each time point was calibrated using the ratio of the same area at 0 h after the end of serum starvation, which was set at 100.

### Time-lapse analysis of Fucci-expressing astrocytes

#### Exposure to NaAsO_2_ and live imaging

NaAsO_2_ (Wako Pure Chemical Industries, Osaka, Japan) was dissolved in sterile-filtered water (Sigma-Aldrich) at a concentration of 100 mM. The NaAsO_2_ solution (100 mM) was further diluted with culture medium to obtain the indicated concentrations. Primary cultured Fucci-expressing astrocytes were subjected to serum starvation (see Section Fluorescence Microscopy of Fucci-Expressing Astrocytes) to synchronize the cell cycle. After synchronization, the cells were exposed to NaAsO_2_ in culture medium at concentrations of 0, 1, 2, or 4 μM. Immediately after starting NaAsO_2_ exposure, astrocytes were placed in an incubation chamber (Tokai Hit, Shizuoka, Japan) equipped to the BioZero 8100 fluorescence microscope. In the incubation chamber, the temperature was controlled at 37°C and the CO_2_ concentration was maintained at 5%. Time-lapse fluorescence imaging began at 1 h and ended at 73 h after initiation of NaAsO_2_ exposure. Fluorescence images of mKO2 and mAG expression, and bright field images were captured every 2 h. The digital image data were obtained from three regions (0.58 mm^2^/region, 1.74 mm^2^ in total) that were randomly selected in each culture dish compartment. Live imaging of Fucci-expressing astrocytes was performed in six primary cultures derived from different animals.

#### Image analysis

We analyzed the intensity of mKO2 and mAG signal emissions, the number of cells expressing mKO2 and mAG, and the cell fate after S phase entry using the digital image data from live imaging. Image analyses were performed using the BZ-II analyzer software. The images were modified to remove the background signal and change the RGB colors of mKO2 and mAG to monochromatic colors. The modified images were used for analysis as described below.

The monochromatic color images of mKO2 (red) and mAG (green) were merged at each time point to measure the intensities of mKO2 and mAG in the same region, which were obtained by measuring the brightness of red and green signals, respectively. The mAG/mKO2 emission ratio was then calculated by dividing the brightness value of the green signal by that of the red signal. The mAG/mKO2 emission ratio at each time point was calibrated using the ratio of the same area at 5 h after NaAsO_2_ exposure, which was set at 100.

To count cells expressing mKO2 and mAG, we used the monochromatic color images for each fluorescent protein, which were obtained every 8 h from 1 h after initiation of NaAsO_2_ exposure. After counting the number of mKO2- and mAG-expressing cells, the value at each time point was then calibrated using the number of cells in the same area at 1 h after NaAsO_2_ exposure, which was set at 100.

To analyze cell fate after S phase entry, the digital images of mKO2, mAG, and bright field, which were obtained from astrocytes with or without exposure to 4 μM NaAsO_2_, were merged at each time point. The merged images at all-time points were then saved as a movie file to observe temporal changes in the expression of mKO2 and mAG, and the morphology of target cells. Target cells were astrocytes that expressed mAG at 41 h in the control group and at 9 h in the NaAsO_2_-exposed group after initiation of NaAsO_2_ exposure, because the population of mAG-expressing cells at these time points was the largest during live imaging for each group. These cells were traced until the end of live imaging to determine their viability. Live cells were defined as cells that expressed mAG followed by expression of mKO2 with normal morphology. Dead cells were defined as cells that expressed mAG followed by loss of fluorescent signals with abnormal morphology. A total of 434 cells in the control group and 426 cells in the NaAsO_2_-exposed group were followed up to determine their cell fate. For each group, the total cell number was defined as 100%, and the percentages of live and dead cell populations were calculated.

### Analysis of GFAP gene copy numbers

#### Exposure to NaAsO_2_ and DNA extraction

Astrocytes originating from the cerebral cortex of C57BL/6J mice were incubated in culture medium containing NaAsO_2_ at concentrations of 0, 1, 2, or 4 μM for 73 h. The astrocytes were then rinsed in Dulbecco's phosphate-buffered saline without calcium and magnesium, and collected in microcentrifuge tubes by scraping the culture plates filled with the buffer. After centrifugation (10,000 rpm, 10 min), the resulting cell pellet was subjected to extraction of total DNA with a DNeasy blood and tissue kit (Qiagen, Hilden, Germany). DNA samples were obtained from eight independent primary cultures derived from different animals.

#### Real-time PCR

Real-time PCR was performed using a LightCycler 96 (Roche Diagnostics, Mannheim, Germany). To prepare standard samples for the GFAP gene, a partial fragment of the mouse GFAP gene (430 bp from 13,801 to 14,230 nt) was cloned by insertion of the DNA fragment into the pCR2.1-TOPO vector supplied in a TOPO TA cloning kit (Invitrogen). The plasmid vector containing a copy of the GFAP gene was serially diluted with nuclease-free water at 3.2 × 10^4^, 8.0 × 10^3^, 2.0 × 10^3^, 5.0 × 10^2^, and 1.25 × 10^2^ copies per 2 μl. Two microliters of the standards and unknown samples containing total DNA extracted from the cultured cells were amplified in a 20 μl reaction mixture containing 200 nM of each primer (forward: 5′-TCCTTTCCACCTCCGCTAAC-3′; reverse: 5′-GTTGGGTCTTGCCTGTCTTC-3′) and 10 μl of 2 × SYBR Premix Ex Taq (Takara Bio, Otsu, Japan). Real-time PCR conditions were initial activation of Taq polymerase for 30 s at 95°C, followed by 40 cycles of 5 s at 95°C for denaturation, and then 30 s at 60°C for annealing and extension with a temperature transition rate of 20°C/s. After real-time PCR, melting curve analysis was carried out to demonstrate the specificity of the PCR product (estimated amplicon size: 138 bp), resulting in a melting curve with a single peak (data not shown). After measurement of the GFAP gene copy number in each sample, the values were expressed as a percentage of the value obtained from cells without NaAsO_2_ exposure, whose viability was set at 100%.

### Apoptosis assay

Primary cultured astrocytes originating from the cerebral cortex of C57BL/6J mice were subjected to serum starvation (see Section Fluorescence Microscopy of Fucci-Expressing Astrocytes) to synchronize the cell cycle and then incubated in culture medium containing NaAsO_2_ at concentrations of 0, 2, or 4 μM for 72 h. The effects of NaAsO_2_ on DNA fragmentation was examined using a Cell Death Detection ELISA Plus Assay kit (Roche Diagnosis) in accordance with the manufacturer's protocol. This assay was performed using six independent primary cultures derived from different animals. The amount of nucleosomes generated from DNA fragmentation is expressed as relative to that in the controls, which was set at 100% for each culture derived from the same animal.

### Analysis of mRNA levels

#### Exposure to NaAsO_2_, RNA extraction, and reverse transcription

Primarily cultured astrocytes were subjected to serum starvation (see Section Fluorescence Microscopy of Fucci-Expressing Astrocytes) to synchronize the cell cycle and then exposed to 0 or 4 μM NaAsO_2_ in culture medium for 9 and 41 h. After exposure, total RNA was extracted and purified with an RNeasy Mini kit (Qiagen) in accordance with the manufacturer's protocol. For each sample, total RNA (650 ng) was reverse transcribed into cDNA in a final reaction volume of 20 μl using a PrimeScript RT Reagent kit (Takara Bio) in accordance with the manufacturer's protocol.

#### Real-time PCR

To measure the gene expression levels of molecules involved in unscheduled S phase entry and apoptosis, p21, p53, E2F1, E2F4, and Gm36566, real-time PCR was performed using a LightCycler 96 (Roche Diagnostics). Equal amounts of cDNA from each sample were combined together and serially diluted with EASY dilution (Takara Bio) to prepare standard samples for each gene. One microliter of standards and diluted unknown samples were amplified in a 10 μl reaction mixture containing 5 μl of 2 × SYBR Premix Ex Taq (Takara Bio) and 100 nM of each primer for specific genes (see Table 1). The real-time PCR settings were the same as those described in Section Real-Time PCR. The amounts of the mRNAs for target genes in each sample were normalized to the mRNA level of the housekeeping gene cyclophilin B (CPB) in the same sample. The normalized mRNA level of each target gene was expressed relative to that in the controls, which was set at 100%. The percentage values were averaged from five different primary cultures.

**Table 1:** **Primer sequences used for real-time PCR**.

	**Forward primer sequence (5′–3′)**	**Reverse primer sequence (5′–3′)**	**NCBI reference sequence**
p21	TAGCTCCTTCCCTGGGATTC	ATAGCAAAGGGGCAGAAAAG	AF035683
p53	GCTTCTCCGAAGACTGGATG	GTCCATGCAGTGAGGTGATG	AB020317
Gm36566	TCCCATTCCCCTATCTGTGT	GCTATTCTCTGCTCCGATCC	XM_011247417
E2F1	ACTGTGACTTTGGGGACCTG	CAGAGGGTATGGATCGTGCT	L21973
E2F4	CTGGCACTTGTGACTGTGCT	AGCACCACCCTCTCTCTGAA	NM_148952
CPB	AGACTGTTCCAAAAACAGTGGA	GATGCTCTTTCCTCCTGTGC	M60456

### Statistical analysis

Two-way factorial analysis of variance (ANOVA) for repeated measures was used to examine the effects of NaAsO_2_ and time on the mAG/mKO2 emission ratio. One-way ANOVA was performed to determine the difference among groups with respect to the number of cells expressing mAG and mKO2, the copy number of the GFAP gene, and the amount of nucleosomes generated from DNA fragmentation. When significant overall effects were detected by one-way ANOVA, the Tukey–Kramer test was used for *post-hoc* analysis. Two-way ANOVA was performed to determine the effects of concentrations and exposure time of NaAsO_2_ on the mRNA levels of target genes. Differences in the populations of live and dead cells between groups with or without exposure to NaAsO_2_ were analyzed by the nonparametric Mann–Whitney *U*-test.

## Results

### Immunoreactivity of GFAP in cultured cells

**Figure 1:**
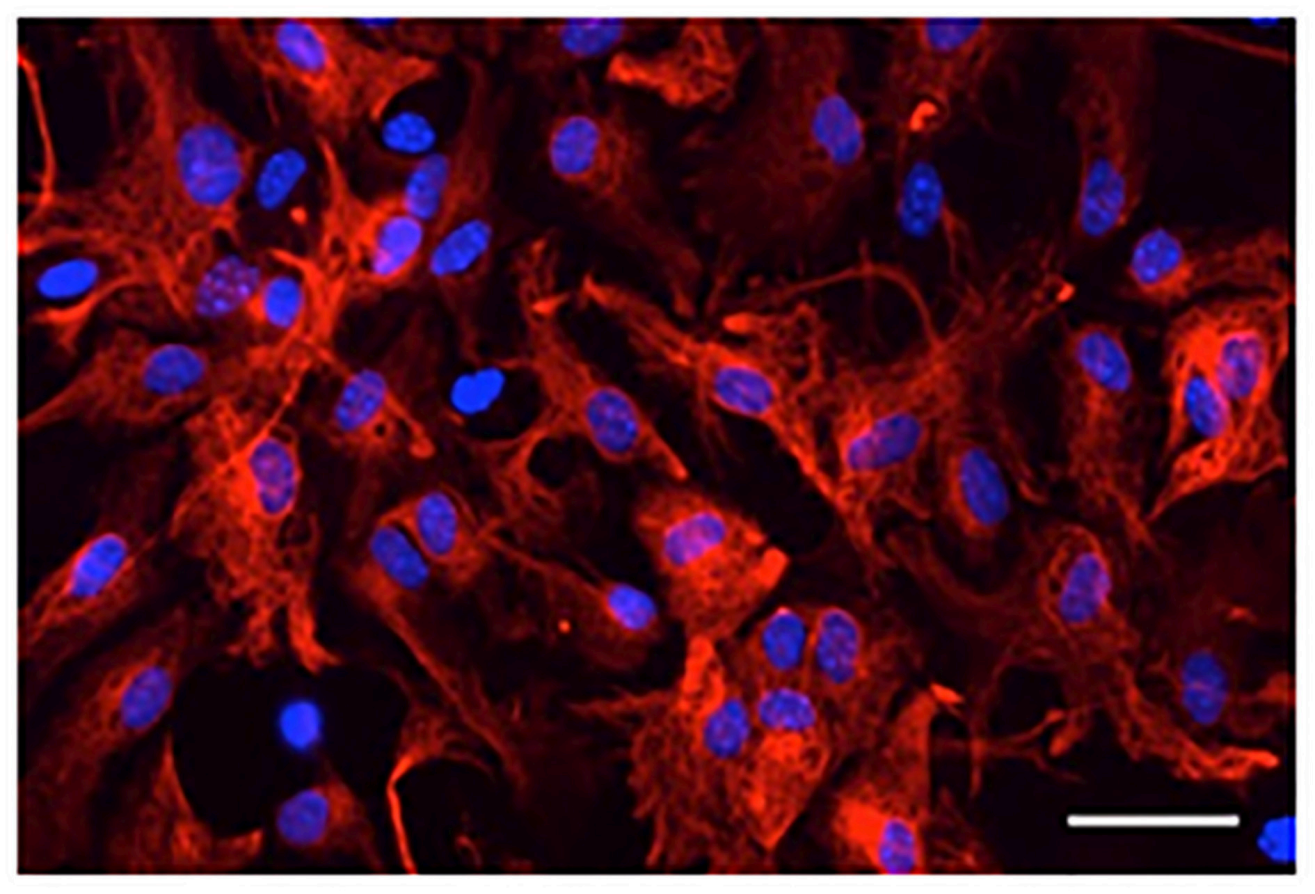
**Digital photomicrograph of primary cultured astrocytes**. Astrocytes were immunostained for GFAP (red) and counterstained with DAPI (blue). Scale bar: 50 μm.

Microscopy and counting DAPI-stained cells with or without GFAP immunoreactivity showed that most DAPI-stained cells exhibited GFAP-immunoreactive signals (98 ± 0.4% in four primary cultures derived from different animals; Figure 1). Thus, cells that were isolated from the mouse brains and cultured *in vitro* were astrocytes expressing GFAP.

### Cell cycle distribution of Fucci-expressing astrocytes

**Figure 2:**
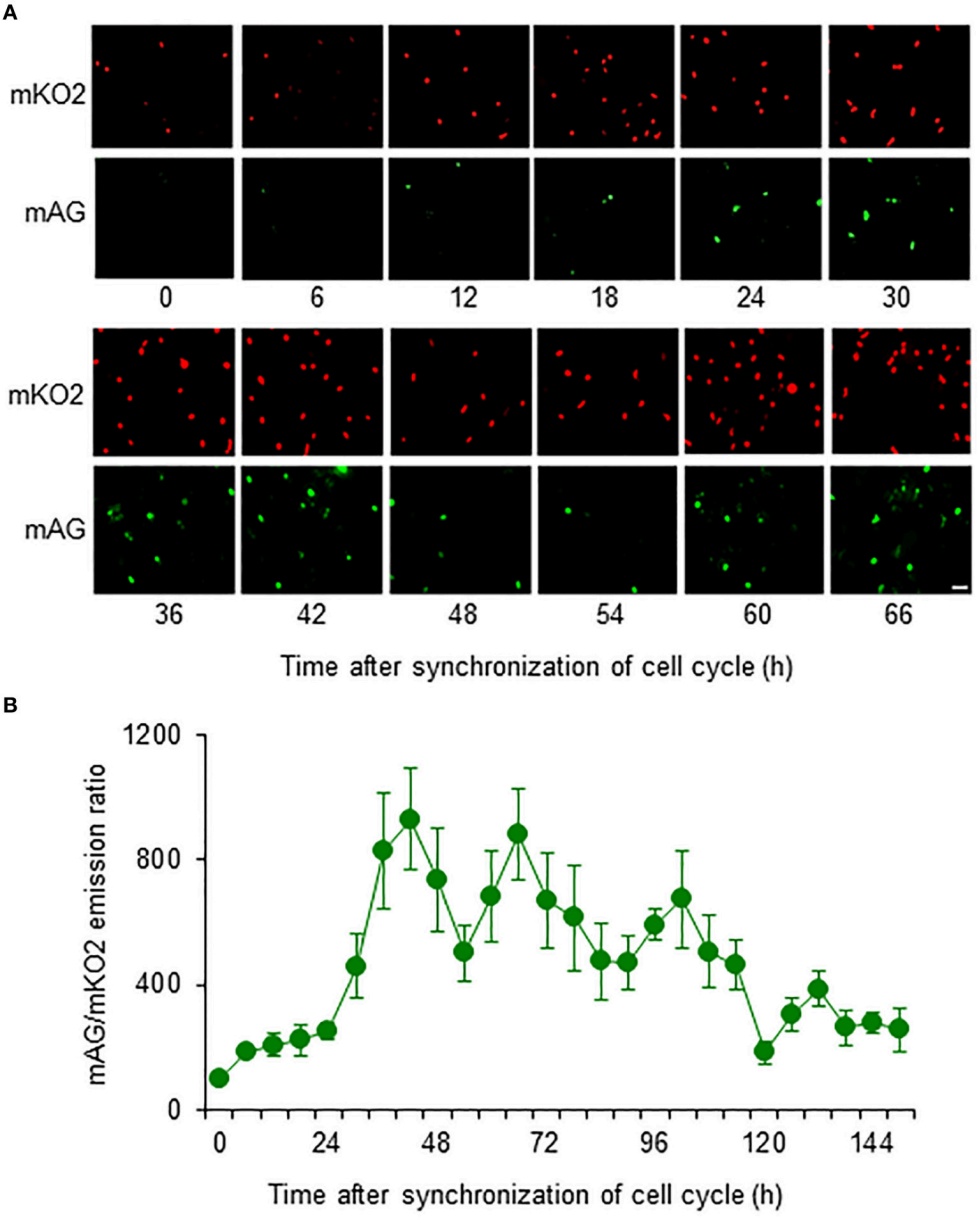
**Temporal changes in mAG and mKO2 emission signals of Fucci-expressing astrocytes**. After astrocytes were serum starved for 72 h to synchronize the cell cycle, they were cultured in medium containing 10% FBS and applied to cell cycle monitoring. **(A)** Time-lapse images of mAG and mKO2 emission signals after the original fluorescent colors were converted to pseudocolors (mAG, green; mKO2, red). Scale bar: 50 μm. **(B)** Temporal changes in the mAG/mKO2 emission ratio of Fucci-expressing astrocytes. All data points are the means ± standard error of the mean (SEM) of four primary cultures derived from different animals. The mAG/mKO2 emission ratio at each time point was calibrated using the ratio at 0 h after the cells were cultured in 10% FBS-containing medium, which was set at 100.

Most primary cultured astrocytes from the cerebral cortex of Fucci tg mice expressed mKO2 after their cell cycles were synchronized by serum starvation (Figure 2A). mAG-expressing astrocytes emerged at 12 h after synchronization of the cell cycle. Forty-two hours after synchronization was the first time that expression of mAG reached a peak. mAG expression then decreased and the second peak of mAG expression occurred at 66 h after synchronization. This cycle of mAG expression was repeated during fluorescence microscopic observation.

The emission ratio of mAG to mKO2 in Fucci-expressing astrocytes changed over time (Figure 2B). The mAG/mKO2 emission ratio was low until 12–18 h after synchronization of the cell cycle. The ratio dramatically increased from 24 to 42 h after synchronization of the cell cycle and then decreased until 54 h after synchronization. The ratio increased again and reached a peak at 66 h after synchronization of the cell cycle in four primary cultures derived from different animals. The cyclic changes in the mAG/mKO2 emission ratio were observed four times during the analysis period. The mean interpeak interval of the mAG/mKO2 emission ratio for each culture was 30, 28, 32, and 30 h. When these values were represented as the cell cycle duration in each culture, the mean of four different experiments was 30 ± 0.82 h, indicating that the cell cycle duration of Fucci-expressing astrocytes is ~30 h.

### Effects of NaAsO_2_ on the cell cycle of astrocytes

**Figure 3:**
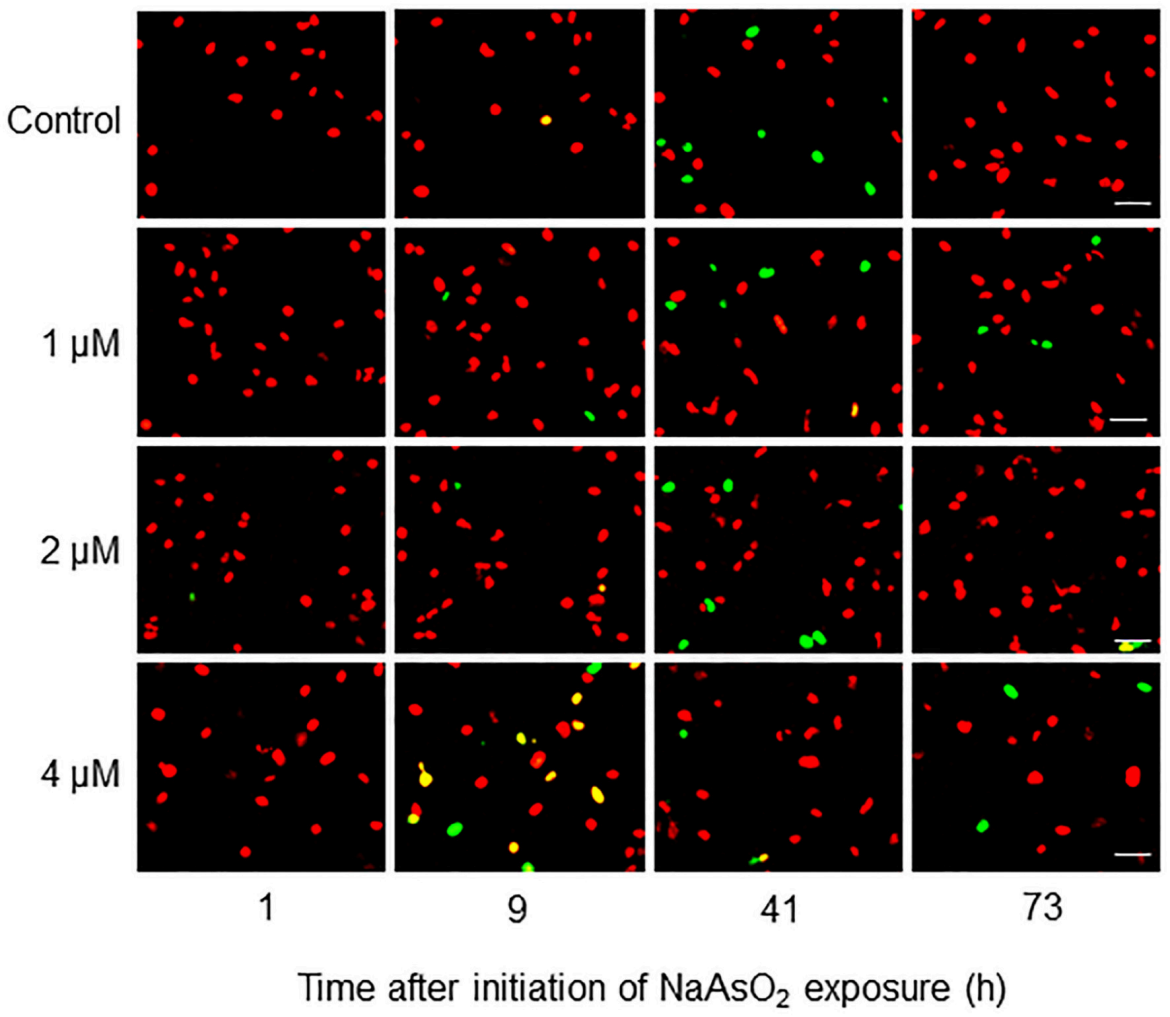
**Time-lapse images of Fucci-expressing astrocytes after initiation of NaAsO_2_ exposure**. Images of mAG and mKO2 signals were merged after the original colors were converted to pseudocolors (mAG, green; mKO2, red). Images were obtained from Fucci-expressing astrocytes exposed to NaAsO_2_ at concentrations of 0 (control), 1, 2, or 4 μM for 1–73 h. Scale bar: 50 μm.

Fucci-expressing astrocytes mostly emitted mKO2 fluorescence signals at the beginning of live imaging with or without NaAsO_2_ exposure, while little emission of mAG signals was observed (Figure 3; Supplementary Movies 1–4). In control and NaAsO_2_ (1 and 2 μM)-exposed groups, astrocytes emitting mAG fluorescence signals were frequently observed between 33 and 49 h after initiation of NaAsO_2_ exposure, which then decreased over time. On the other hand, in astrocytes exposed to 4 μM NaAsO_2_, mAG fluorescence signals were found at 5–9 h after NaAsO_2_ exposure. In some astrocytes exposed to 4 μM NaAsO_2_, the fluorescent emissions of mAG and mKO2 had disappeared after 31 h of exposure to NaAsO_2_.

**Figure 4:**
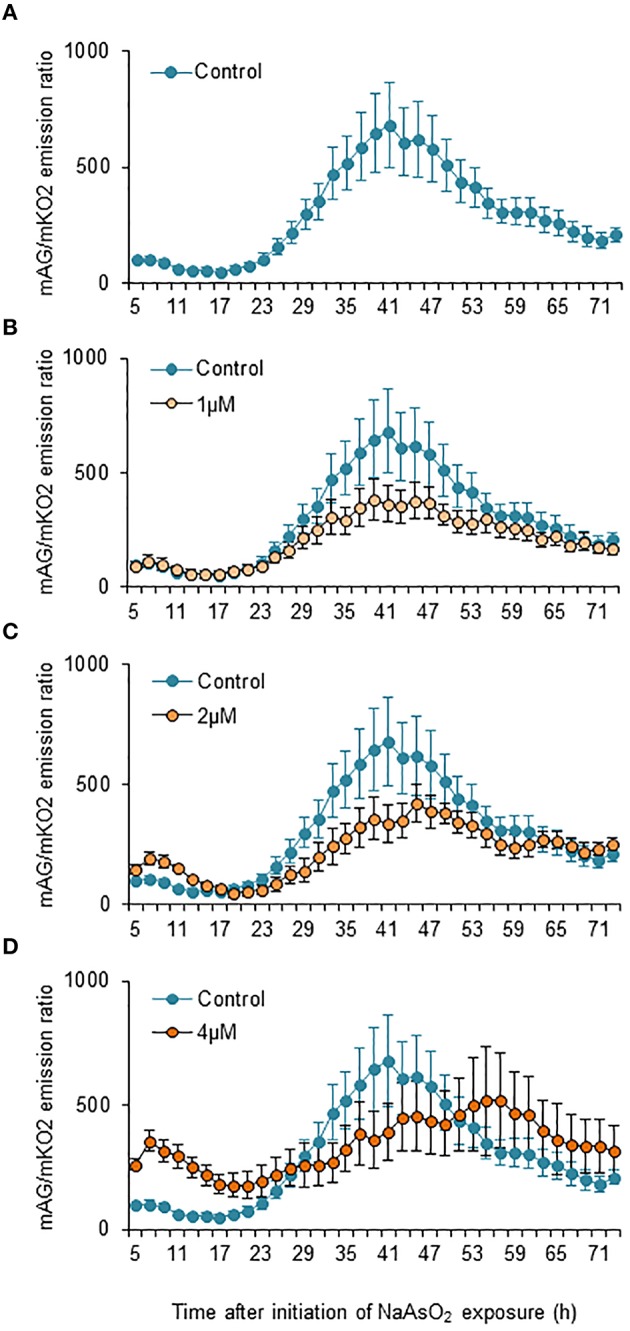
**Effects of NaAsO_2_ exposure on the mAG/mKO2 emission ratio of Fucci-expressing astrocytes**. Temporal changes in the mAG/mKO2 emission ratio of the control group **(A)** and groups exposed to NaAsO_2_ at 1 μM **(B)**, 2 μM **(C)**, or 4 μM **(D)** from 5 to 72 h after initiation of NaAsO_2_ exposure. Significant effects of the concentration and time of NaAsO_2_ exposure and the interaction between the concentration and time on the ratio were found in two-way ANOVA for repeated measures. The ratio at each time point was calibrated using the ratio at 5 h after NaAsO_2_ exposure, which was set at 100. All data are the means ± SEM of six primary cultures derived from different animals.

Two-way ANOVA for repeated measures indicated that the temporal changes in the mAG/mKO2 emission ratio differed significantly among groups [*F*_(3, 175)_ = 49.3, *p* < 0.05 × 10^−26^], over time [*F*_(34, 175)_ = 2.91, *p* < 0.000005], and by interactions between the main factors [*F*_(102, 525)_ = 1.84, *p* < 0.00001]. In control and NaAsO_2_ (1 and 2 μM)-exposed groups, the mAG/mKO2 emission ratio was low and maintained a stable level until 23 h after NaAsO_2_ exposure, which then increased over time and reached a peak at 39–45 h after NaAsO_2_ exposure, followed by a gradual decrease until the end of live imaging (Figure 4). The peak levels of the mAG/mKO2 emission ratio in NaAsO_2_ (1 and 2 μM)-exposed groups were lower than those in the control group. The temporal change in the mAG/mKO2 emission ratio of the NaAsO_2_ (4 μM)-exposed group was different from that of other groups. In the NaAsO_2_ (4 μM)-exposed group, a peak of the mAG/mKO2 emission ratio was found at 7 h after initiation of NaAsO_2_ exposure, whereas no obvious peak in the ratio was observed when the ratio showed a peak in the control and NaAsO_2_ (1 and 2 μM)-exposed groups.

### Effects of NaAsO_2_ on the number of Fucci-expressing astrocytes

**Figure 5:**
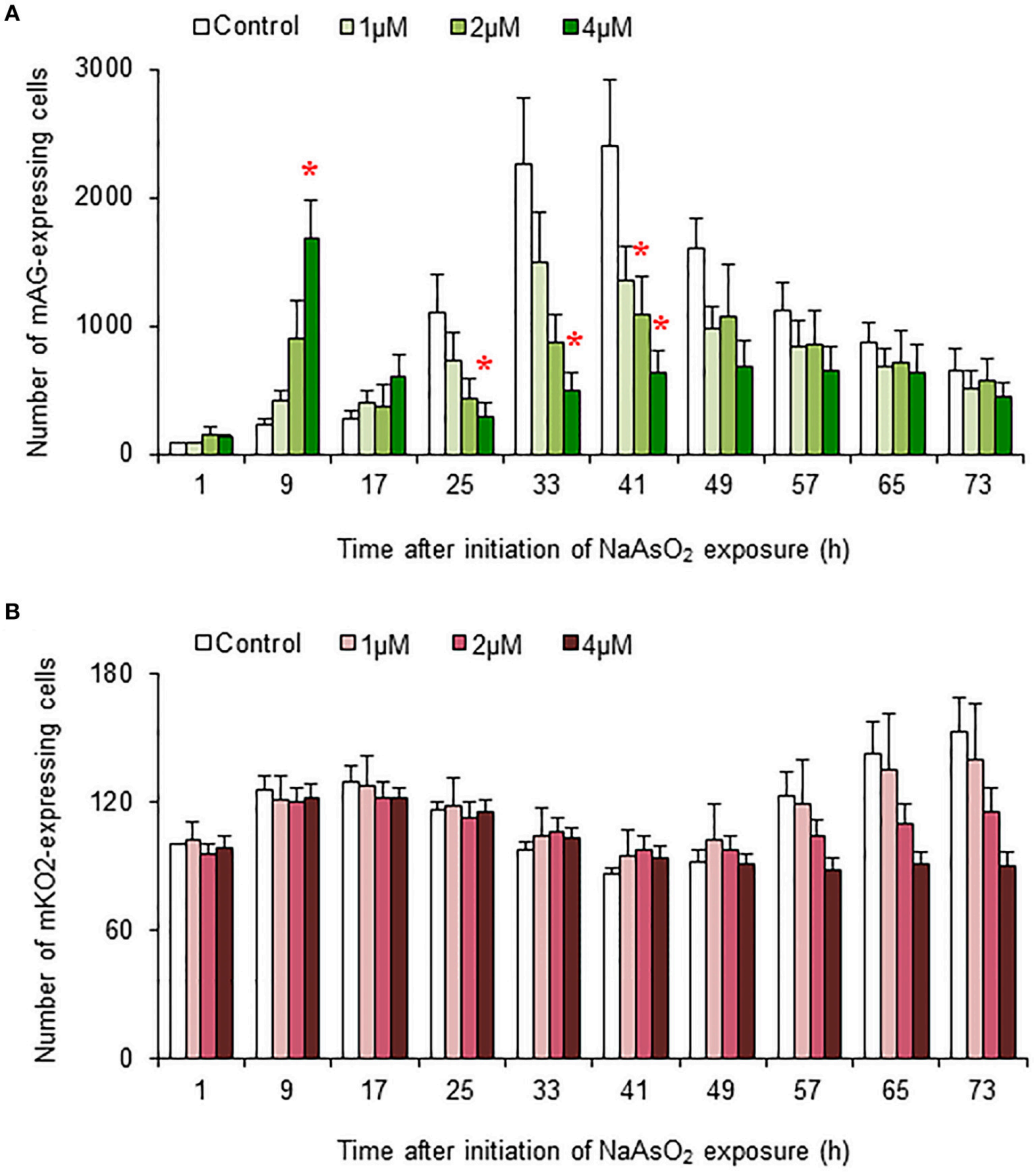
**Effects of NaAsO_2_ exposure on the number of Fucci-expressing astrocytes**. The number of cells expressing mAG **(A)** and mKO2 **(B)** at the indicated time point after initiation of NaAsO_2_ exposure. The value for each time point was calibrated with the number of cells in the control group at 1 h after NaAsO_2_ exposure, which was set at 100. All data are the means ± SEM of six primary cultures derived from different animals. ^*^p < 0.05 vs. control at the same time point.

In control and NaAsO_2_ (1 μM)-exposed groups, the numbers of mAG-expressing cells at 33 and 41 h after NaAsO_2_ exposure were larger than those at other time points for each group (Figure 5A). Compared with the control group, the number of mAG-expressing cells was significantly (*p* < 0.05) smaller at 41 h after NaAsO_2_ exposure in the NaAsO_2_ (2 μM)-exposed group and at 25, 33, and 41 h after NaAsO_2_ exposure in the NaAsO_2_ (4 μM)-exposed group. In the NaAsO_2_ (4 μM)-exposed group, the number of mAG-expressing cells was highest at 9 h after NaAsO_2_ exposure, which was significantly (*p* < 0.05) larger than that in the control group.

There was tendency toward an increase in the number of cells expressing mKO2 from the beginning to the end of live imaging in control and NaAsO_2_ (1 μM)-exposed groups (Figure 5B). At 57, 65, and 73 h after NaAsO_2_ exposure, the total number of mKO2-expressing cells showed a concentration-dependent decrease, although there was no significant difference in the number of mKO2-expressing cells among the groups at each time point.

### Effects of NaAsO_2_ on cell death after S phase entry

**Figure 6:**
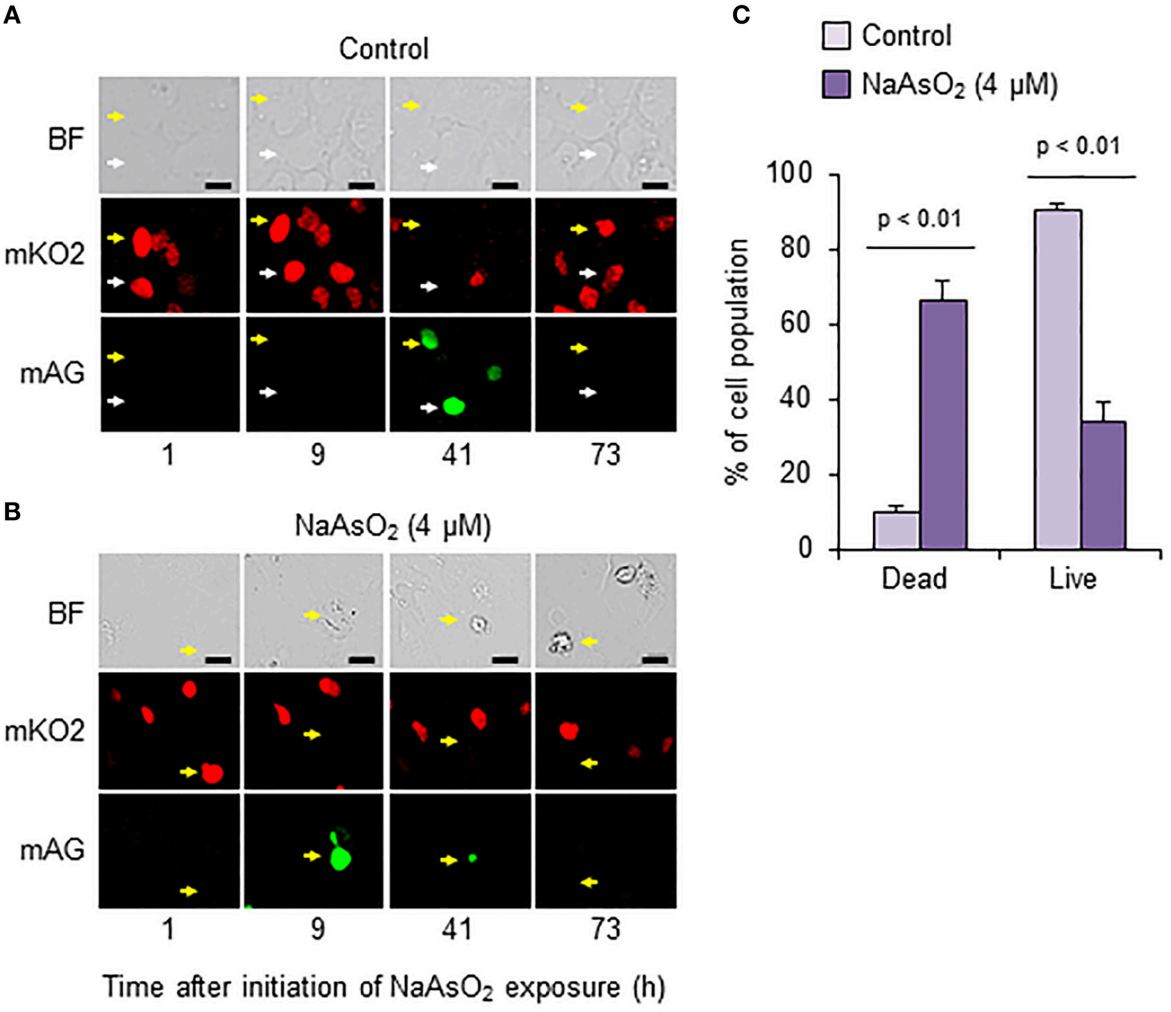
**Effects of NaAsO_2_ exposure on cell fate after S phase entry**. Digital photomicrograph of Fucci-expressing astrocytes captured with bright field (BF) and emission of mKO2 and mAG after pseudocolor conversion from the original fluorescent colors (mKO2, red; mAG, green) in control and NaAsO_2_ (4 μM)-exposed groups **(A,B)**. Yellow and white arrows indicate the same cell or area. Scale bar: 20 μm. Cell population after Fucci-expressing cells entered S phase **(C)**. The percentage values of dead and live cells after S phase entry were calculated with the total number of cells that entered S phase, which was set at 100%. Values are the means ± SEM of six primary cultures derived from different animals.

To analyze cell fate after S phase entry, we traced Fucci-expressing astrocytes that emitted mAG fluorescence signals at 41 h in the control group and at 9 h after initiation of NaAsO_2_ exposure in the NaAsO_2_ (4 μM)-exposed group. As a result, most cells in the control group were alive at the end of live imaging, because the fluorescent emission signals changed from mAG to mKO2, and the morphology was normal (Figure 6A). Conversely, in most astrocytes of the NaAsO_2_ (4 μM)-exposed group, the emission signal of mAG, which was observed at 9 h after NaAsO_2_ exposure, had disappeared by the end of live imaging (Figure 6B). In addition, the cells did not express mKO2 and exhibited abnormal morphology with a debris-like structure. The population of live cells after S phase entry was significantly (*p* < 0.01) larger in the control group than in the NaAsO_2_ (4 μM)-exposed group (Figure 6C). In contrast, the population of dead cells after S phase entry was significantly (*p* < 0.01) larger in the NaAsO_2_-exposed group than in the control group.

### Effects of NaAsO_2_ on the copy number of the GFAP gene and DNA fragmentation

**Figure 7:**
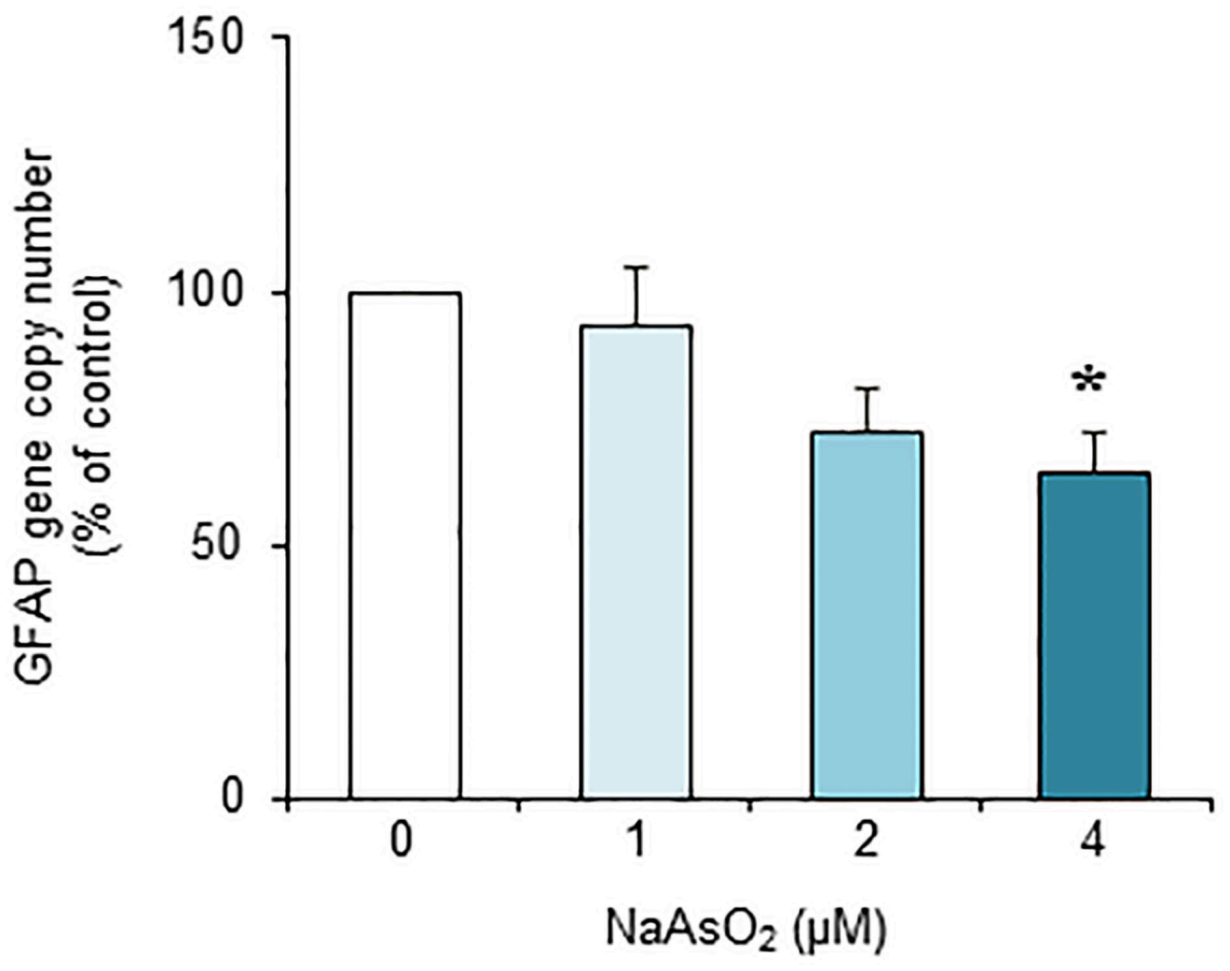
**Effects of NaAsO_2_ exposure on the copy number of the GFAP gene**. The GFAP gene copy number is presented as a percentage of the control. Values are the means ± SEM of eight primary cultures derived from different animals. ^*^p < 0.05 vs. control.

Exposure to NaAsO_2_ for 73 h significantly [*F*_(3, 28)_ = 4.30, *p* < 0.05] reduced the GFAP gene copy number in a concentration-dependent manner (Figure 7). The copy number of the GFAP gene in the NaAsO_2_ (4 μM)-exposed group was significantly (*p* < 0.05) smaller than that in the control group. We found no significant effect of lower NaAsO_2_ concentrations.

**Figure 8:**
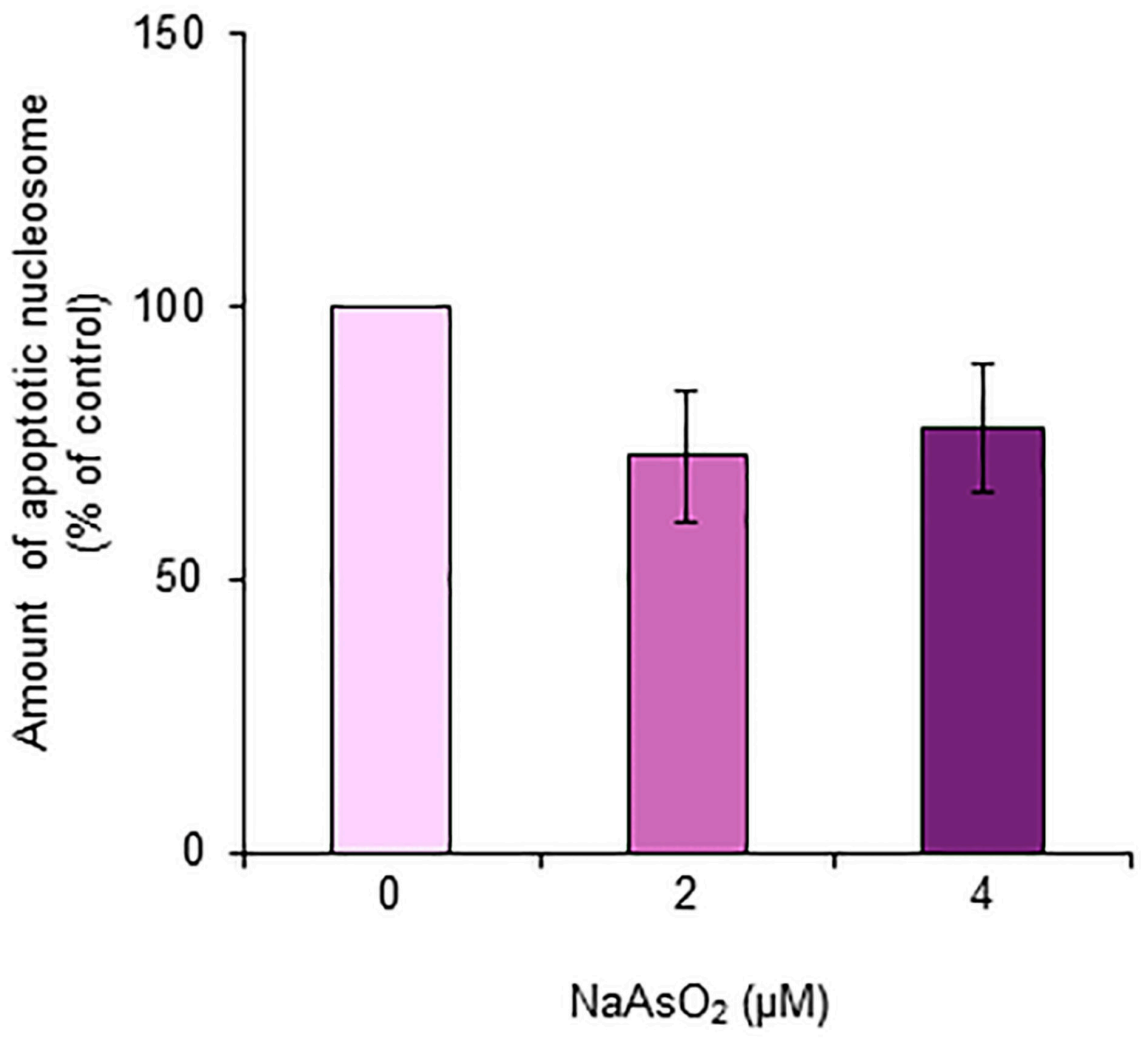
**Amount of apoptotic nucleosomes in astrocytes**. The amount of apoptotic nucleosomes was assayed in astrocytes exposed to NaAsO_2_ (0, 2, and 4 μM) and is represented as a percentage of the control. Values are the means ± SEM of six primary cultures derived from different animals.

The amount of nucleosomes generated from DNA fragmentation in astrocytes did not significantly [*F*_(1, 15)_ = 2.21, *p* = 0.14] change after exposure to NaAsO_2_ (2 and 4 μM) for 72 h (Figure 8).

### Effect of NaAsO_2_ on the mRNA expression of apoptotic markers and cell cycle regulators

**Figure 9:**
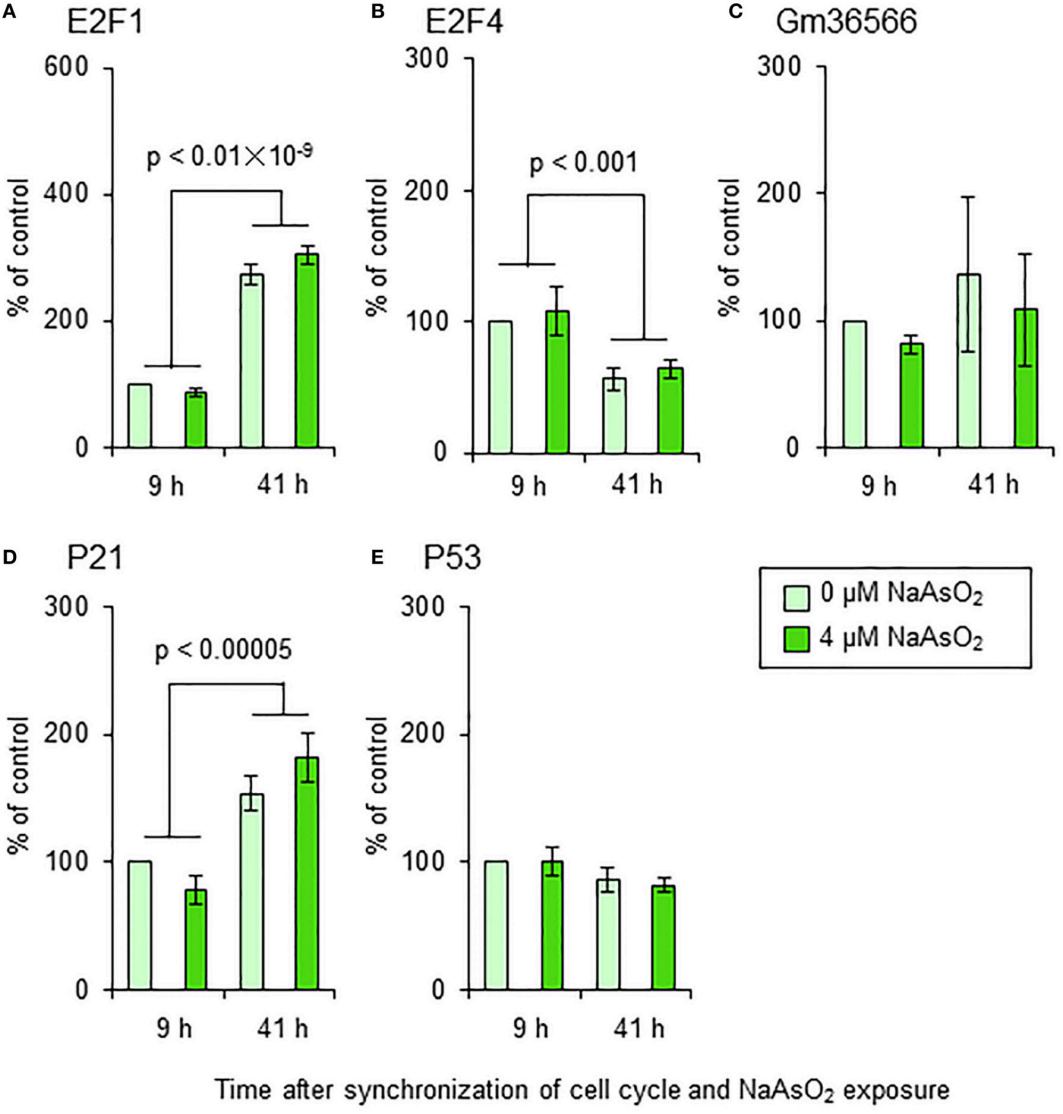
**mRNA levels of E2F1 (A), E2F4 (B), Gm36566 (C), p21 (D), and p53 (E) in astrocytes**. Astrocytes were exposed to NaAsO_2_ (0 and 4 μM) for 9 and 41 h. The mRNA level of each gene was normalized to that of CPB and are expressed relative to that of the control (0 μM for 9 h), which was set at 100%. Values are the means ± SEM of five primary cultures derived from different animals.

We did not find any significant effects of NaAsO_2_ exposure on the mRNA levels of E2F1, E2F4, Gm36566, p21, or p53 in astrocytes (Figure 9). The mRNA levels of E2F1, E2F4, and p21, but not Gm36566 and p53, changed over time after synchronization of the cell cycle of astrocytes. The mRNA levels of E2F1 [*F*_(1, 16)_ = 300.69, *p* < 0.01 × 10^−9^] and p21 [*F*_(1, 16)_ = 37.74, *p* < 0.00005] were significantly higher at 41 h than 9 h after synchronization of the cell cycle, while the mRNA levels of E2F4 [*F*_(1, 16)_ = 16.28, *p* < 0.001] were higher at 9 h than 41 h after synchronization.

## Discussion

There is serious concern about contamination of ground water with arsenic, because chronic consumption of arsenic-contaminated water causes an impairment of cognitive functions (Calderon et al., 2001; Tsai et al., 2003; Wasserman et al., 2007). The World Health Organization recommends a limit of 0.01 mg/L (0.01 ppm) arsenic in water (World Health Organization, 2011). However, arsenic contamination in tube well water was found in Bangladesh at a concentration of more than 0.3 mg/L (0.3 ppm; Smith et al., 2000). In a native Andean population living in a part of Argentina, where drinking water contains arsenic at about 0.2 mg/L (0.2 ppm), the concentrations of arsenic were 9 μg/L in cord blood, 11 μg/L in maternal blood, 34 μg/kg in placenta, and 2.3 μg/kg in breast milk (0.009, 0.011, 0.034, and 0.0023 ppm, respectively; Concha et al., 1998a,b). According to a study measuring the accumulated levels of arsenic in newborn mice of mothers that were chronically exposed to NaAsO_2_ (10–80 ppm) during the gestational period via drinking water, the accumulated level of arsenic in the brain was higher than that in the liver and blood, and ranged from ~100 to 700 ng/g (0.1–0.7 ppm; Markowski et al., 2011). We previously reported that NaAsO_2_ at 0.5–10 μM induced cell death and suppressed neuritogenesis of cultured neurons (Koike-Kuroda et al., 2010; Aung et al., 2013; Maekawa et al., 2013). In this study, to examine whether NaAsO_2_ at the micromolar concentrations affect cultured astrocytes, we set the exposure concentrations of NaAsO_2_ at 1, 2, and 4 μM (equivalent to 0.129, 0.258, and 0.516 ppm, respectively), which were similar to environmental pollution levels and higher than human exposure levels by ~200-times or less.

It is known that cultured cells undergo phototoxic damage induced by frequent illumination with excitation light under a fluorescence microscope (Cervinka et al., 2008). Our previous study revealed that serum supplementation (the combination of 1% horse serum and 0.5% FBS) is vital to protect PC12 cells from phototoxic damage and enables live cell imaging without phototoxic damage (Koike-Kuroda et al., 2010). On the other hand, the doubling time of primary cultured astrocytes from the rat cerebral cortex is 6 days under the culture condition of 2% fetal calf serum (Geisert et al., 1996). This finding indicates that culturing astrocytes with serum supplementation at low concentrations is not beneficial for live imaging to monitor the cell cycle of astrocytes, because it requires a longer time (more than 6 days), which would increase the risk of phototoxic damage. Therefore, to promote cell proliferation and shorten the doubling time of astrocytes, we performed live imaging analysis of primary cultured Fucci-expressing astrocytes under the culture condition of 10% FBS. The duration of the cell cycle in Fucci-expressing astrocytes subjected to live imaging corresponded to that determined by fluorescence microscopy, suggesting that the live imaging technique in our current study could monitor the cell cycle without severe phototoxic damage.

Live imaging analysis of Fucci-expressing astrocytes showed that NaAsO_2_ exposure significantly altered the cell cycle. The cell population entering S phase, when the cell population had reached to a peak level in the control group without NaAsO_2_ exposure, was decreased by NaAsO_2_ in a concentration-dependent manner. In contrast, the cell population entering unscheduled S phase at 9 h after initiation of NaAsO_2_ exposure was increased by NaAsO_2_ in a concentration-dependent manner. The findings in our current study suggest that NaAsO_2_ exposure disrupts the cell cycle and forces astrocytes to enter S phase at an unscheduled timing. In particular, 4 μM NaAsO_2_ had significant effects to disrupt cell cycle regulation and induce unscheduled S phase entry. Moreover, we traced the morphology as well as mKO2 and mAG expression of Fucci-expressing astrocytes to determine the fate of astrocytes that underwent unscheduled S phase entry induced by 4 μM NaAsO_2_. As a result, more than 60% of astrocytes that entered S phase at 9 h after NaAsO_2_ exposure had died, with disappearance of fluorescent mKO2 and mAG signals, and transforming debris-like structures until the end of live imaging. We also measured the copy number of the GFAP gene at 73 h after NaAsO_2_ exposure, and found a significant decrease in the gene copy number induced by 4 μM NaAsO_2_. Taken together, it appears likely that NaAsO_2_ exposure at 4 μM decreases the number of astrocytes by inducing unscheduled S phase entry-coupled cell death. There is a report showing a slight increase in the viability of cultured rat cerebellar astrocytes by NaAsO_2_ exposure at 1 μM for 24 h, but higher concentrations (5–50 μM) decrease the cell viability (An et al., 2016). In contrast, there is a report indicating that the cell viability is unaffected even when cultured rat astrocytes are exposed to 0.3 mM arsenite for 8 h, but it is decreased at 24 h after exposure (Koehler et al., 2014). Although the detailed mechanisms are largely unknown, arsenic may have dual effects to increase or decrease cell viability, which are dependent on the concentration and exposure time. Our current study supports the notion that arsenic decreases the viability of cultured astrocytes, and suggests that decreased cell viability is due to the reduction of cell number induced by unscheduled S phase entry-coupled cell death.

It is well-known that unscheduled S phase entry is linked to apoptotic cell death after DNA damage (Dimova and Dyson, 2005; Cho and Liang, 2011). The live imaging analysis in our current study showed that the mAG/mKO2 emission ratio and the number of mAG-expressing cells increased faster with NaAsO_2_ exposure, indicating that up-regulation of geminin expression at an unscheduled timing is induced by NaAsO_2_ exposure. It has been reported that overexpression of geminin induces apoptotic cell death (Shreeram et al., 2002). In this context, we speculated that apoptosis is responsible for the unscheduled S phase entry-coupled cell death induced by NaAsO_2_ exposure. To test this hypothesis, we examined the effects of NaAsO_2_ on the amount of nucleosomes generated from DNA fragmentation in astrocytes. However, in contrast to our expectations, we did not find any significant effect of NaAsO_2_ on the amount of nucleosomes generated from DNA fragmentation. In addition, we examined whether NaAsO_2_ exposure affected the expression levels of certain molecules involved in unscheduled S phase entry and apoptosis. We measured the mRNA levels of p53, a principal regulator of apoptosis (Fridman and Lowe, 2003; Cho and Liang, 2011), E2F1, an S phase gene transcriptional activator and programed cell death inducer (Hou et al., 2000; Pardee et al., 2004; Dimova and Dyson, 2005; Lazzerini Denchi and Helin, 2005; Cho and Liang, 2011), E2F4, an S phase gene transcriptional suppressor (Dimova and Dyson, 2005), p21, a cyclin dependent kinase inhibitor (Cho and Liang, 2011) and unscheduled S phase inducer (Bedelbaeva et al., 2010), and Gm36566, the mouse ortholog of Killin, which acts as a S-phase-coupled apoptosis regulator (Cho and Liang, 2011). Considering the results of the apoptosis assay with reference to the amount of nucleosomes, the gene expression of these molecules was not significantly affected by NaAsO_2_ exposure, although the mRNA levels of E2F1, E2F4, and p21 had temporally changed with or without NaAsO_2_ exposure. Taken together, the unscheduled S phase entry that occurred in astrocytes after NaAsO_2_ exposure may be induced without alteration of the expression of these molecules. In addition, the subsequent cell death may be caused by mechanisms other than apoptosis. However, the mechanisms were not determined in this study. Further studies are needed to clarify the molecular mechanisms of arsenic toxicity, which induce unscheduled S phase entry-coupled cell death in astrocytes.

In summary, we examined the effects of NaAsO_2_ exposure on the cell cycle, viability, and apoptotic cell death of cultured mouse cerebral astrocytes. The results of these analyses suggest that 4 μM NaAsO_2_ significantly induces unscheduled S phase entry that is coupled with cell death by mechanisms other than apoptosis.

## Author contributions

ST and FM designed the study; NH, HS, KS, and KA performed live imaging analyses; NH, HS, and SM performed molecular analyses; NH and ST wrote the manuscript; FM and MT critically revised the manuscript.

## Funding

This study was supported by Grants-in-Aid for Scientific Research from the Ministry of Education, Culture, Sports, Science and Technology of Japan (23310043 and 15K14556 to ST; 24590307, 15K08223, and 15K14556 to FM), grants from the National Institute for Environmental Studies [1011AF005 and 1416AT001] and a grant from the National Center for Child Health and Development (25-3) to FM.

### Conflict of interest statement

The authors declare that the research was conducted in the absence of any commercial or financial relationships that could be construed as a potential conflict of interest.
